# Handgrip strength in older adults from Antioquia-Colombia and comparison of cutoff points for dynapenia

**DOI:** 10.1038/s41598-023-28898-1

**Published:** 2023-01-31

**Authors:** Fredy Alonso Patiño-Villada, Alejandro Estrada-Restrepo, Juan Aristizábal

**Affiliations:** 1 Physiscal Activity for Health Research Group, Institute of Physical Education, University of Antioquia, Medellín, 050034 Colombia; 2 Demography and Health Research Group, School of Nutrition and Dietetics, University of Antioquia, Medellín, 050034 Colombia; 3 Physiology and Biochemistry Research Group-PHYSIS, Faculty of Medicine, University of Antioquia, Medellín, 050034 Colombia; 4 School of Nutrition and Dietetics, University of Antioquia, Medellín, 050034 Colombia

**Keywords:** Geriatrics, Nutrition, Public health

## Abstract

Handgrip strength is a predictor of functional impairment and presence of morbimortality in older adults. However, appropriate reference values and cutoff points are required for its optimal use. This study describes handgrip characteristics in the older adult population of Antioquia-Colombia and compares the dynapenia handgrip cutoffs proposed for Colombians with international criteria. A cross-sectional study including 1592 older adults was done. Dynapenia prevalence by handgrip was analyzed using the following cutoffs: European Consensus of Sarcopenia (2018), Asian Working Group for Sarcopenia (2019), Chilean (2018), and Colombian (2019). Handgrip strength significantly decreased with aging, showing a positive and strong association with functional and health parameters. The highest prevalence of dynapenia was found with the Asian Consensus cutoffs (26.1%) and the lowest with the Colombian cutoffs (0.8%). Low agreement was found between the Colombian cutoffs with the European Consensus (kappa = 0.059; *p* < 0.001), the Asian Consensus (kappa = 0.039; *p* < 0.001) and the Chilean proposal (kappa = 0.053; *p* < 0.001). Dynapenia using the Chilean, European, and Asian cutoffs was associated with physical inactivity, presence of multimorbidity, slow gait speed, nutritional risk, and low calf circumference. Meanwhile, the Colombian cutoffs was only associated with slow gait speed and low calf circumference. The handgrip cutoffs proposed for Colombians seems to underestimate the dynapenia prevalence in older people from Antioquia. Furthermore, these cutoff points did not show associations with relevant functional and health parameters. The handgrip cutoffs proposed for Colombians should be used with caution.

## Introduction

Worldwide older adult population has significantly increased in recent years and is expected to continue growing. In 2019, one of nine people in the world was 65 years or older. This proportion is projected to increase to be one out of six by 2050^1^. In 2015, the World Health Organization (WHO) highlighted the need to promote and maintain older adults’ physical and mental capacities^2^. For achieving this goal, health professionals require simple evaluation methods to measure and monitor the medical-nutritional status of geriatric population. The assessment of muscle strength using manual dynamometry, or handgrip, has a predictive power for cognitive impairment, slow mobility, low functional condition, and mortality in older adults^3,4^. Likewise, handgrip is used in diagnosing sarcopenia, frailty, and malnutrition^5–7^. For these reasons, manual dynamometry is proposed as a clinical indicator that should be included in medical and nutritional status assessments^8,9^.

The optimal use of handgrip as a health indicator requires appropriate reference values and cutoff points to classify low grip strength (dynapenia), and it is recommended that these parameters are derived from the very population to be evaluated^5,10^. The European Consensus for the Definition and Diagnosis of Sarcopenia (EWSOP2) has updated dynapenia cutoffs in 2018^5^. The Asian Working Group for Sarcopenia defined cutoffs for this population group in 2019^11^. In North America, reference values and cutoffs were developed from the National Nutrition and Health Study in 2016^12^. In South America, countries like Chile (2018) and Brazil (2020) have also published reference values^13,14^. Multicentric studies, including data from Colombia, have proposed cutoffs for older people^15,16^. Noteworthy is the fact that cutoffs proposed for Colombian population in 2019 are the first borderline values developed using a nationally representative sample with data from the SABE study (Survey on Health, Well-Being, and Aging in Latin America and the Caribbean)^17^. However, these Colombian cutoffs are twenty percent lower, or even more, than those recommended in Europe^5^, Asia^11^, and Chile^13^. Their application could underestimate the prevalence of sarcopenia since low handgrip strength, according to EWSOP2^5^ is one the first criteria in the diagnostic of this syndrome. The reasons for the low Colombians cutoffs remain unclear, although they may seem to be related with the characteristics of the reference population conditions (e.g., ethnic, social, cultural, or lifestyle) and the statistical method applied for derivation.

Therefore, it is necessary to evaluate the cutoffs proposed for Colombians in 2019 and their association with indicators of multimorbidity and functionality in a Colombian population sample different from the SABE study. Accordingly, the first objective of this study is to describe the handgrip characteristics in older population from Antioquia, Colombia. The second objective is to compare the dynapenia prevalence in this population using the international cutoffs with those originally proposed for Colombians in 2019^17^ and alternative Colombian borderline values corresponding to the 25th percentile of the population described by Ramirez et al.^17^. The third objective is to analyze the variations in the dynapenia associations with functional and health parameters after applying the above-mentioned cutoffs.

## Results

### Characteristics of the study population

Out of the 1592 older adults evaluated, 59.0% (n = 913) were women. The mean age was 70.1 ± 7.7 years, 70.5 ± 7.8 years in men, and 69.8 ± 7.7 years in women. 89% percent of the participants had up to primary school studies as their maximum educational level (Table 1). The median for body weight was 63.7 kg (IQR: 55.6–72.2), for height 154.5 cms (IQR: 148.6–161.4), for calf circumference 34.6 cms (IQR: 32.5–37.1), and for BMI 26.2 kg/m^2^ (IQR: 23.4–29.6). Comparatively, men were heavier (men: 66.8 kg, IQR: 59.6–76.4; women: 62.9 IQR: 53.6–71.2; *p* < 0.001), taller (men:163.0 cms, IQR: 158.3–168.6; women 150.2 cms, IQR: 144.7–154.6; *p* < 0.001), and had lower body mass index (BMI) (men: 25.4 kg/m^2^, IQR: 23.1–28.5; women: 27.9 kg/m^2^, IQR:24.6–31.3; *p* < 0.001). Men and women presented similar calf circumference median values (34.6 cm for men and 34.5 cm for women; *p* = 0.046). In women, physical inactivity, multimorbidity, slow gait speed, risk of malnutrition, low calf circumference classification, and excess body weight shown higher percentage (Table 1).

**Table 1:** Distribution of handgrip strength and demographics, health, functionality, and anthropometrics characteristics by sex.

Variable	Total			Men			Women		
	n	%	Median (IQR)	n	%	Median (IQR)	n	%	Median (IQR)
Antioquia	1592	100	24.2 (19.7–30.9)	679	41.0	32.2 (27.7–36.8)	913	59.0	21.0 (17.8–24.3)
Age^a^									
60–64	485	31.7	25.6^a^ (20.6–34.6)	198	30.0	34.6^a^ (32.2–38.4)	287	33.0	22.6^a^ (18.7–25.4)
65–69	358	19.4	25.5^a^ (20.4–31.9)	157	19.3	33.4^a,b^ (29.8–37.8)	201	19.4	21.4^a^ (18.3–24.7)
70–74	299	20.8	24.8^a^ (20.7–29.3)	124	19.0	30.7^b^ (28.6–36.0)	175	22.0	21.9^a,b^ (19.7–24.8)
75–79	212	15.3	23.1^a,b^ (19.1–29.3)	91	18.9	28.4^c^ (23.0–31.0)	121	12.8	20.3^b,c^ (15.9–23.3)
80–84	140	6.6	21.0^b,c^ (16.5–27.0)	62	7.1	28.0^c,d^ (22.5–35.1)	78	6.3	18.9^c,d^ (14.6–21.6)
≥ 85	98	6.2	19.7^c,d^ (16.2–21.1)	47	5.7	23.2^d,e^ (18.2–30.9)	51	6.5	17.9^d^ (14.9–20.5)
p*			< 0.001			< 0.001			< 0.001
Educational level^a^									
No education/Preschool	917	47.3	24.2^a^ (19.6–30.3)	392	48.6	30.9^a^ (27.0–35.6)	525	46.4	20.7^a^ (17.0–24.4)
Primary school	514	42.1	23.4^a^ (19.7–30.1)	206	39.3	32.6^a,b^ (27.6–37.8)	308	44.1	21.1^a,b^ (17.8–23.8)
Secundary school	98	6.1	30.7^b^ (22.4–34.6)	56	7.8	34.6^b^ (31.6–38.9)	42	4.9	22.5^b^ (19.1–26.4)
College	60	4.6	24.9^a,b^ (21.5–32.3)	24	4.4	33.2^a,b^ (29.2–40.3)	36	4.7	23.2^a,b^ (20.1–24.2)
p*			< 0.001			0.002			0.023
Physical activity									
Active	1013	63.3	25.7 (20.7–33.0)	519	75.7	32.8 (28.0–37.0)	494	54.7	21.4 (18.8–24.6)
Inactive	569	36.7	22.7 (17.7–27.7)	155	24.3	30.3 (27.1–34.6)	414	45.3	20.4 (16.3–23.4)
p**			< 0.001			< 0.001			< 0.001
Multimorbidity									
No	861	54.1	25.8 (21.0–32.6	431	64.5	32.6 (28.9–37.0)	430	46.9	21.5 (18.8–24.4)
Yes	731	45.9	22.9 (18.5–28.9)	248	35.5	31.4 (26.5–35.6)	483	53.1	20.1 (16.5–23.9)
p**			< 0.001			0.0001			0.0002
Gait speed									
Normal (≥ 0.8 m/s)									
846		55.0	26.6 (21.4–34.5)	423	62.8	34.6 (30.1–38.2)	423	49.8	22.4 (19.2–24.7)
Slow (< 0.8 m/s)									
685		45.0	22.5 (17.7–27.3)	224	37.2	29.3 (23.0–33.5)	461	50.2	20.1 (15.9–23.9)
p**			< 0.001			< 0.001			< 0.001
Mini nutritional assessment									
Normal									
881		63.8	25.6 (20.8–32.5)	411	70.9	32.6 (28.1–37.0)	470	58.6	21.4 (19.0–24.9)
Risk/Malnutrition									
513		36.2	23.1 (17.8–29.3)	197	29.1	30.3 (27.1–35.6)	316	41.4	20.3 (15.9–23.4)
p**			< 0.001			0.001			< 0.001
Calf circumference									
Adequate (≥ 31 cm)									
1257		86.6	25.4 (20.4–32.2)	575	92.8	32.6 (28.6–37.0)	682	82.1	21.4 (17.8–24.8)
Low (< 31 cm)									
167		13.4	21.2 (17.2–24.3)	47	7.2	26.2 (22.4–29.3)	120	17.9	19.7 (15.3–22.7)
p**			< 0.001			< 0.001			< 0.001
Body mass index^a^									
Undernutrition	312	20.5	24.2^a,b^ (20.5–32.2)	166	25.4	32.1^a^ (27.7–34.7)	146	17.1	20.5^a^ (16.7–22.7)
Normal	637	43.0	24.8^a^ (20.6–31.7)	308	52.9	31.3^a,b^ (27.3–36.7)	329	36.2	21.4^a,b,c^ (18.7–24.4)
Overweight	380	24.4	23.5^a,b^ (17.9–29.1)	143	17.4	33.6^b^ (28.8–37.9)	237	29.3	20.1^b,c^ (17.5–25.0)
Obesity	214	12.1	22.8^b^ (19.3–27.2)	36	4.4	34.1^a,b^ (29.7–40.3)	178	17.5	21.4^c^ (18.7–25.0)
p*			0.0242			0.0042			0.0001

Median (IQR): median (percentile 25—percentile 75). *Kruskal Wallis test. Multiple comparison Dunn’s post-hoc test **U Mann Whitney test.

^a^Different letters in same column indicate significant differences between groups.

### Handgrip and health/functional parameters

Handgrip was higher in apparently healthier older adults (Table 1). Handgrip was higher (*p* < 0.05) among active people, with gait speed ≥ 0.8 m/s, calf circumference ≥ 31 cms, adequate BMI, and without multimorbidities or nutritional risk. These results were similar in men and women, except for the BMI classification in men, in which those with excess body weight showed the highest handgrip value (Table 1).

Differences in dynapenia prevalence were found using the cutoff points of interest (Table 2). The highest prevalence was found with the Asian Consensus (26.1%) and the lowest prevalence with the original Colombian cutoffs (0.8%). Tables 2 and 3 show the associations between health status, functionality parameters, and anthropometric characteristics with dynapenia classifications. Dynapenia classifications using the Chilean, the European, the Asian and the 25th percentile as alternative Colombian borderline cutoffs showed associations with physical inactivity, presence of multimorbidity, slow gait speed, nutritional risk, and low calf circumference. Meanwhile, dynapenia classification with the original Colombian cutoffs was associated with slow gait speed and low calf circumference variables.

**Table 2:** Health, functional and anthropometrics characteristics according to dynapenia by European, Asian consensus and Chilean cutoffs.

Variable		European consensus (EWGSOP2) 2018		Asian consensus 2019		Chileans 2018	
		Dynapenia	No dynapenia	Dynapenia	No dynapenia	Dynapenia	No dynapenia
		% (95% CI)	% (95% CI)	% (95% CI)	% (95% CI)	% (95% CI)	% (95% CI)
Antioquia	1592	18.4 (15.3–21.9)	81.6 (78.1–84.7)	26.1 (22.4–30.1)	73.9 (69.9–77.6)	20.3 (17.1–23.9)	79.7 (76.1–82.9)
Physical activity							
Active	1013	51.1 (41.2–60.9)	66.0 (60.9–70.8)	54.8 (46.2–63.0)	66.3 (60.8–71.4)	53.8 (44.6–62.8)	65.7 (60.5–70.6)
Inactive	569	48.9 (39.1–58.8)	34.0 (29.2–39.1)	45.2 (37.0–53.8)	33.7 (28.6–39.2)	46.2 (37.2–55.4)	34.3 (29.4–39.5)
p*		0.008		0.023		0.026	
Multimorbidity							
No	861	40.7 (31.3–50.9)	57.2 (52.5–61.7)	44.6 (36.0–53.5)	57.5 (52.6–62.3)	42.5 (33.7–51.9)	57.1 (52.3–61.7)
Yes	731	59.3 (49.1–68.7)	42.8 (38.3–47.5)	55.4 (46.5–64.0)	42.5 (37.7–47.4)	57.5 (48.1–66.3)	42.9 (38.3–47.7)
p*		0.004		0.012		0.006	
Gait speed							
Normal (≥ 0.8 m/s)	846	25.6 (19.2–33.3)	61.4 (56.5–66.2)	33.8 (25.7–42.9)	62.3 (57.1–67.3)	29.1 (22.5–36.7)	61.4 (56.4–66.2)
Slow (< 0.8 m/s)	685	74.4 (66.7–80.8)	38.6 (33.8–43.5)	66.2 (57.1–74.3)	37.7 (32.7–42.9)	70.9 (63.3–77.5)	38.6 (33.8–43.6)
p*		< 0.001		< 0.001		< 0.001	
Mini nutritional assessment							
Normal	881	50.5 (39.7–61.2)	66.8 (61.1–72.1)	52.3 (42.6–61.8)	67.9 (62.1–73.2)	52.2 (42.2–62.1)	66.8 (60.9–72.1)
Risk/Malnutrition	513	49.5 (38.8–60.3)	33.2 (27.9–38.9)	47.7 (38.2–57.4)	32.1 (26.8–37.9)	47.8 (37.9–57.8)	33.2 (29.7–39.1)
p*		0.008		0.006		0.013	
Calf circumference							
Adequate (≥ 31 cm)	1257	77.1 (69.1–83.5)	88.8 (83.0–92.8)	79.7 (73.3–84.9)	89.1 (82.6–93.3)	77.4 (70.0–83.4)	89.0 (83.1–93.0)
Low (< 31 cm)	167	22.9 (16.5–30.9)	11.2 (7.2–17.0)	20.3 (15.1–26.7)	10.9 (6.7–17.4)	22.6 (16.6–30.0)	11.0 (7.0–16.9)
p*		0.010		0.030		0.010	
Body mass index							
Undernutrition	312	22.2 (16.1–29.7)	20.1 (15.9–25.0)	21.0 (16.0–27.1)	20.3 (15.8–25.6)	23.5 (17.5–30.6)	19.7 (15.5–24.8)
Normal	637	44.4 (34.4–54.9)	42.7 (37.8–47.7)	41.0 (32.8–49.8)	43.6 (38.5–48.9)	44.3 (35.0–53.9)	42.6 (37.7–47.8)
Overweight	380	24.8 (15.6–36.9)	24.4 (20.7–28.4)	29.0 (20.1–39.9)	22.9 (19.7–26.4)	24.5 (16.0–35.5)	24.4 (20.7–28.5)
Obesity	214	8.6 (5.5–13.3)	12.9 (10.7–15.5)	8.9 (6.2–12.8)	13.2 (10.9–16.0)	7.8 (5.0–12.0)	13.2 (10.9–15.9)
p*		0.622		0.306		0.351	

European consensus EWGSOP2 2018: European Working Group on Sarcopenia in Older People update in 2018^5^. Asian consensus 2019^11^. Chileans 2018: Reference values of handgrip dynamometry in older Chileans^13^.

*Maximum likelihood estimation.

**Table 3:** Health, functionality, and anthropometrics characteristics according to dynapenia by Colombian proposal.

Variable	n	Original Colombian cutoffs 2019		Alternative Colombian borderlines 2019 (< p25)	
		Dynapenia	No dynapenia	Dynapenia	No dynapenia
		% (95% CI)	% (95% CI)	% (95% CI)	% (95% CI)
Antioquia	1592	0.8 (0.4–1.8)	99.2 (98.2–99.6)	5.5 (4.2–7.1)	94.5 (92.9–95.8)
Physical activity					
Active	1013	32.5 (9.4–69.1)	63.6 (59.0–67.9)	44.7 (32.0–58.1)	64.4 (59.6–68.9)
Inactive	569	67.5 (30.9–90.6)	36.4 (32.1–41.0)	55.3 (41.9–68.0)	35.6 (31.1–40.4)
p*		0.089		0.006	
Multimorbidity					
No	861	60.5 (25.5–87.3)	54.1 (49.8–58.3)	38.3 (26.0–52.3)	55.1 (50.6–59.4)
Yes	731	39.5 (12.7–74.5)	45.9 (41.7–50.2)	61.7 (47.7–74.0)	44.9 (40.6–49.4)
p*		0.731		0.024	
Gait speed					
Normal (≥ 0.8 m/s)	846	14.2 (2.2–55.1)	55.4 (50.9–59.8)	28.7 (18.2–42.1)	56.5 (51.8–61.1)
Slow (< 0.8 m/s)	685	85.8 (44.9–97.8)	44.6 (40.2–49.1)	71.3 (57.9–81.8)	43.5 (38.9–48.2)
p*		0.017		< 0.001	
Mini nutritional assessment					
Normal	881	30.8 (8.5–68.1)	64.1 (59.0–68.9)	46.8 (33.2–60.8)	64.8 (59.5–69.7)
Risk/Malnutrition	513	69.2 (31.9–91.5)	35.9 (31.1–41.0)	53.2 (39.2–66.8)	35.2 (30.3–40.5)
p*		0.069		0.018	
Calf circumference					
Adequate (≥ 31 cm)	1257	50.2 (16.6–83.6)	86.9 (82.3–90.5)	69.2 (54.7–80.6)	87.7 (82.8–91.3)
Low (< 31 cm)	167	49.8 (16.4–83.4)	13.1 (9.5–17.7)	30.8 (19.4–45.3)	12.3 (8.7–17.2)
p*		0.036		0.004	
Body mass index					
Undernutrition	312	48.1 (14.5–83.5)	20.2 (16.6–24.4)	23.5 (13.4–38.0)	20.3 (16.5–24.7)
Normal	637	26.4 (7.3–62.1)	43.1 (38.7–47.7)	48.7 (35.2–62.3)	42.7 (38.0–47.4)
Overweight	380	12.4 (1.6–55.2)	24.5 (21.0–28.4)	20.4 (10.7–35.4)	24.7 (21.0–28.7)
Obesity	214	13.0 (1.7–56.6)	12.1 (10.2–14.4)	7.4 (3.3–15.6)	12.4 (10.4–14.7)
p*		0.389		0.547	

Original Colombian cutoffs 2019: reference cutoffs for handgrip strength among older adults^17^. Alternative Colombian borderlines 2019 (< p25): values lower than the 25-percentile taken from Ramirez-Velez et al.^17^.

*Maximum likelihood estimatio.

### Concordance and agreement of handgrip cutoff points

**Figure 1:**
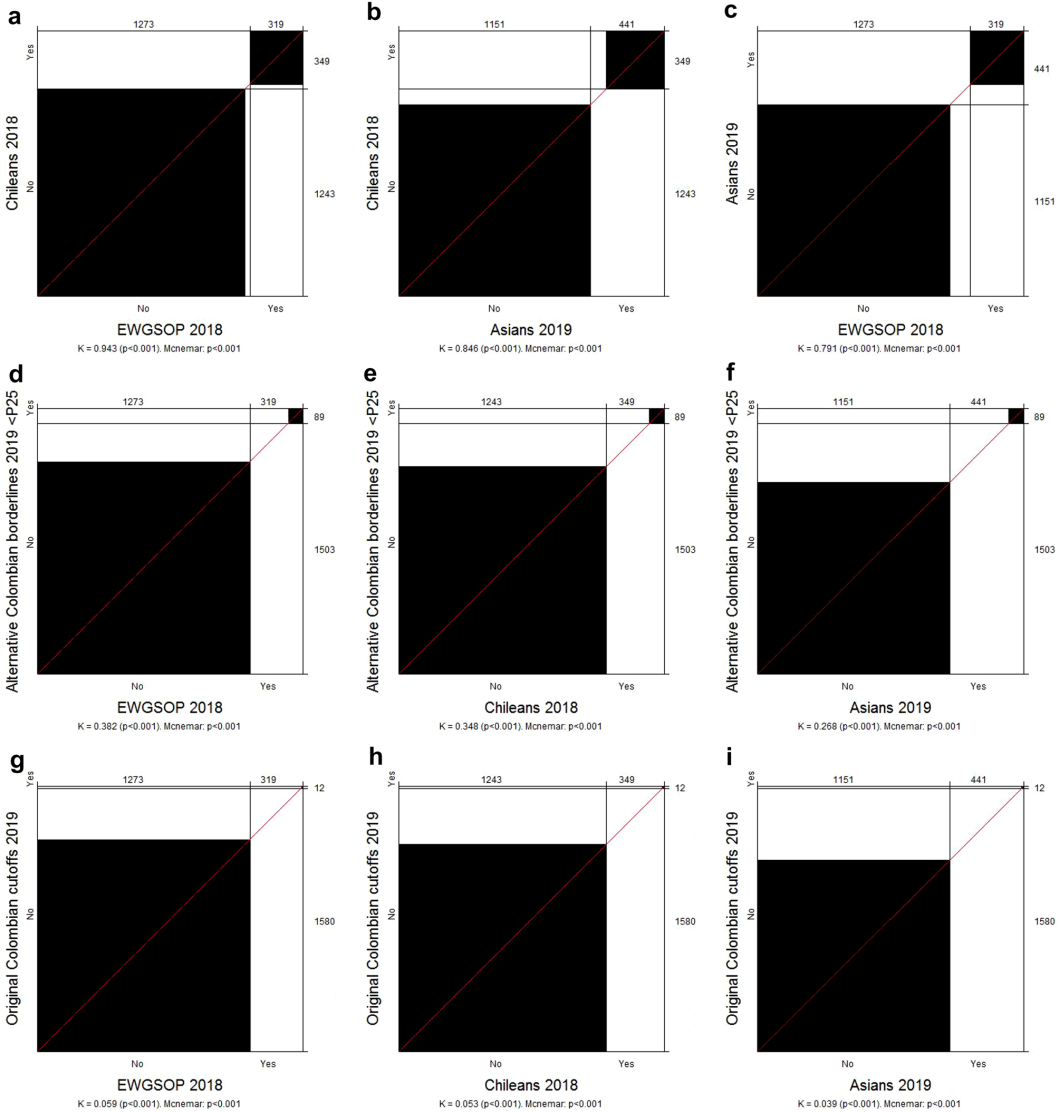
Agreement plot of dynapenia between different cutoff points. European consensus EWGSOP2 2018: European Working Group on Sarcopenia in Older People update in 2018^5^. Asian consensus 2019^11^. Chileans 2018: Reference values of handgrip dynamometry in older Chileans^13^. Original Colombian cutoffs 2019: reference cutoffs for handgrip strength among older adults^17^. Alternative Colombian borderlines2019 (< p25): values lower than the 25-percentile taken from Ramirez-Velez et al.^17^. K: Cohen’s Kappa Coefficient. McNemar: McNemar test. < p25: Values below 25 percentile.

The concordance and agreement between cutoffs for dynapenia classification are shown in Table 4 and Fig. 1. The highest concordance was found between the European Consensus and the Chilean proposal (k = 0.943; *p* < 0.001); the black squares fill almost the entire area of the diagonal rectangles (Fig. 1a). The diagonal line crossing smoothly through the vertex of the rectangles (Fig. 1a) evinces symmetry (minimal bias) between dynapenia classifications using the European Consensus and the Chilean proposal. When dynapenia classification was done using the 25th percentile for alternative borderline values, fair agreements were found with the European Consensus (k = 0.382; *p* < 0.001), the Asian Consensus (k = 0.268; *p* < 0.001) and the Chilean proposal (k = 0.348; *p* < 0.001). The black squares fill a halfway area of the diagonal rectangles (Fig. 1d–f). Slight agreements were found between the original Colombian cutoffs with the European Consensus (k = 0.059; *p* < 0.001), the Asian Consensus (k = 0.039; *p* < 0.001) and the Chilean proposal (k = 0.053; *p* < 0.001); the black squares fill a small area of the agreement cell for dynapenia (upright diagonal rectangles in Fig. 1g–i). Sex disaggregated analysis showed kappa results similar to those using the aggregate population (Supplementary Table S1 online).

**Table 4:** Concordance between different cutoff points for dynapenia.

Dynapenia classification	European consensus (EWGSOP2) 2018		Asian Consensus 2019		Chileans 2018	
	No	Yes	No	Yes	No	Yes
Asian consensus 2019						
No	1151	0				
Yes	122	319				
Kappa	k = 0.791; *p* < 0.001					
McNemar	< 0.001					
Chileans 2018						
No	1243	0	1151	92		
Yes	30	319	0	349		
Kappa	k = 0.943; *p* < 0.001		k = 0.846; *p* < 0.001			
McNemar	< 0.001		< 0.001			
Original Colombian cutoffs 2019						
No	1273	307	1151	429	1243	337
Yes	0	12	0	12	0	12
Kappa	k = 0.059; *p* < 0.001		k = 0.039; *p* < 0.001		k = 0.053; *p* < 0.001	
McNemar	< 0.001		< 0.001		< 0.001	
Alternative Colombian borderlines 2019 (< p25)						
No	1273	230	1151	352	1243	260
Yes	0	89	0	89	0	89
Kappa	k = 0.382; *p* < 0.001		k = 0.268; *p* < 0.001		k = 0.348; *p* < 0.001	
McNemar	< 0.001		< 0.001		< 0.001	

European consensus EWGSOP2 2018: European Working Group on Sarcopenia in Older People update in 2018^5^. Asian consensus 2019^11^. Chileans 2018: Reference values of handgrip dynamometry in older Chileans^13^. Original Colombian cutoffs 2019: reference cutoffs for handgrip strength among older adults^17^. Alternative Colombian borderlines 2019 (< p25): values lower than the 25-percentile taken from Ramirez-Velez et al.^17^.

K: Cohen’s Kappa Coefficient. McNemar: McNemar test. < p25: Values below 25 percentile.

## Discussion

The first objective of this study was to describe handgrip characteristics in the older population from Antioquia, Colombia. In this population, handgrip strength significantly decreases with aging, in both men and women. Handgrip strength has shown a positive and strong association with functional and health parameters. These findings corroborate the potential value of manual dynamometry in medical and nutritional assessments of older population. In relation to the second objective, dynapenia prevalence was below 1% when applying the original Colombian cutoffs^17^. The prevalence increased up to 5.5% using the alternative Colombian borderline values. While applying the international criteria, prevalence was around 20%. Regarding the third objective, dynapenia classification using the cutoffs proposed for Colombians did not show any significant association with physical inactivity, presence of multimorbidity, or malnutrition risk. This suggests that the handgrip cutoff points proposed for Colombians seems to underestimate the prevalence of dynapenia in the older population of Antioquia.

Handgrip strength was higher in subjects that were physically active, morbidity free, and free of malnutrition risk, which is in line with previous studies^18–21^. Likewise, older adults with adequate handgrip strength showed normal gait speed, thus supporting this measurement's utility to identify older people with locomotion impairments^22,23^. These results endorse handgrip strength as a screening and monitoring tool for nutrition and health status in older population^3,4,8^.

Using the original cutoff points proposed for Colombians, the prevalence of dynapenia in the older population of Antioquia was rather low (0.8%); this percentage shows a slight agreement with the international criteria (kappa < 0.06). Probably, the prevalence of dynapenia is higher in Antioquia´s population, as it is suggested by the international cutoff points (between 18.4 and 26.1%) and the health conditions found of multimorbidity (45.9%), risk of malnutrition (36.2%), physical inactivity (36.7%) and slow gait speed (45.0%). Accordingly, the application of the original Colombian cutoffs seem to underestimate the prevalence of sarcopenia, frailty, and malnutrition in this population, which may delay the treatments of these conditions, and then affecting the general wellbeing of the older adult Colombian population.

The low performance of the original handgrip cutoffs proposed for Colombians is probably due to several factors. One factor might relate to the sociodemographic characteristics of the population used for deriving the cutoff points. However, the Colombian older population was similar in terms of age, gender, and educational levels as to the population from Chile, and other developing countries of South America wherein cutoffs were higher^13,14^. Therefore, the methodology used to derive the cutoffs may have played a major role. The proposed handgrip cutoff points were derived from the SABE-Colombia study, which included population aged between 60 and 108^17^. Since handgrip strength decreases with aging and its reduction is related to functional impairment, considering older adults as reference population to derive cutoff points may be inappropriate. EWGSOP2 cutoffs for dynapenia, proposed in 2019 (< 27 kg for men and < 16 kg for women), were derived from British adult population^5^. These cutoff points correspond to a < 2.5 T-score value of the maximum handgrip strength found in adult men (29–39 years old) and women (26–42 years old)^5^. Cutoff points for the older population developed in adults have shown to be helpful in monitoring health parameters like bone mass density^24^.

Using values below one standard deviation for establishing cutoff points could be another factor that contributed to the low performance of the original handgrip thresholds proposed for Colombians. One standard deviation is close to 15th and 16th percentiles on a normal sample distribution, and this could be a low value for handgrip cutoff points in older people. Lera et al.^13^, using a sample of older adult Chilean population, established handgrip cutoffs using the 25th percentile. Lera’s thresholds seem to be more appropriate for the older population of Antioquia, as shown in the results of this study. Similarly, the Asian handgrip cutoff points^11^, developed with older population using the 20th percentile, showed results in accordance with the multimorbidity states and functionality status found in the population of Antioquia.

The alternative Colombian borderlines yielded higher dynapenia prevalence (5.5%) than the cutoffs originally proposed for Colombians in 2019 (0.8%). Dynapenia classification with the alternative Colombian borderlines showed additional associations with physical inactivity, presence of multimorbidity, and nutritional risk evaluated by MNA; such associations were missing when the cutoff points proposed for Colombians were used. From a clinical viewpoint, the application of the alternative Colombian borderlines appears to be more reasonable for diagnosing dynapenia in older people from Antioquia. However, these borderline values generate lower dynapenia prevalence than the international criteria. Therefore, it seems reasonable to continue using the international handgrip cutoff points, especially when using manual dynamometry in health promotion and disease prevention among older population of Antioquia.

A strength of this study was its representative sample of older people from Antioquia, which included people from urban and rural areas. Moreover, this study used a handgrip device and measurement protocol resembling the Colombian SABE survey. One limitation of this study is its cross-sectional design that limits establishing causal conclusions. However, the analysis in this study does not claim this type of association. Rather, the analysis focuses on reporting the characteristics of handgrip strength and the concordance between the different classifications for dynapenia.

## Conclusions

Handgrip strength was higher in men than women, in youngest-old people (60–64 years), in those with normal nutritional status, lacking multimorbidity, and presenting optimal functional indicators. This study found low concordance between the original handgrip cutoffs proposed for Colombians regarding other international criteria. Moreover, the Colombian thresholds did not show any significant associations with physical inactivity, presence of multimorbidity or malnutrition risk. The handgrip cutoffs proposed for Colombians should be used with caution.

## Methods

This is a cross-sectional study derived from the survey Food and Nutritional Profile of Households in Antioquia, 2019 (which stands in Spanish for *“Perfil Alimentario y Nutricional de los Hogares de Antioquia, 2019”*). Antioquia is the second largest department in Colombia and has over six million inhabitants. The Government of Antioquia and the School of Nutrition and Dietetics from the University of Antioquia carried out the survey with strict quality control processes for data collection^25^. Households were selected using a probabilistic, stratified, and multi-stage sampling design. All adults 60 years and older dwelling in the selected households were included in the study. A total of 1592 older people participated in the study, making up a representative sample for residential area (urban–rural). People with physical or mental limitations were excluded from the analysis due to limitations to collect anthropometric and handgrip measurements. The study followed the Helsinki Declaration guidelines. The measurement protocols were approved by the Ethics Committee from Universidad de Antioquia’s Faculty of Medicine (Act number 12, August 23, 2018). Participants voluntarily manifested their consent to participate in the study and signed an informed consent letter.

Trained and standardized health staff performed anthropometric and physical activity measurements. Body weight was measured with an electronic scale (Seca 878, California, United States of America), height with a portable stadiometer (Seca 213, California, United States of America), and calf circumference with a metal tape (Lufkin W6006ME, Texas, United States of America). Each measurement was made twice. A third measurement was done when a difference between measurements was greater than 0.1 kg in body weight, 0.5 cm (cms) in height, or 0.2 cms in calf. Calf circumference values below 31.0 cms were considered low^26^. Handgrip strength was measured twice in each hand using a digital dynamometer (Takei 5401, Tokyo, Japan). A third measurement was performed when a difference ≥ 10% was found between the first and the second measurements. The highest measurement of both hands was used as the maximum handgrip since this value is probably less affected by the number of trials than the average of the measurements^27^.

National and international handgrip cutoffs were used to classify dynapenia (Table 5). The analysis included the cutoffs of the European consensus EWGSOP2 2018^5^, the Asian 2019 consensus^11^, the Chilean 2018 proposal^13^, the Original Colombian 2019 cutoffs^17^ and alternative Colombian borderline values corresponding to the 25th percentile of the population described by Ramirez et al.^17^. This analysis was included by the authors while reviewing alternative borderline values in contrast to the original proposed for Colombians.

**Table 5:** Cuttoff values for handgrip dynamometry to measure dynapenia by European and Asian consensus, Chilean and Colombian proposals.

Cutoffs values	Men	Women
European consensus (EWGSOP2) 2018	< 27 kg	< 16 kg
Asian consensus 2019	< 28 kg	< 18 kg
Chileans 2018	< 28 kg	< 16 kg
Original Colombian cutoffs 2019	60–64 years < 17.4 kg 65–69 years < 15.7 kg 70–74 years < 14.3 kg 75–79 years < 12.3 kg 80–84 years < 10.1 kg 85 years and older < 8.6 kg	60–64 years < 10.1 kg 65–69 years < 8.9 kg 70–74 years < 8.2 kg 75–79 years < 6.7 kg 80–84 years < 5.3 kg 85 years and older < 4.9 kg
Alternative Colombian borderlines 2019 (< p25)	60–64 years < 25.1 kg 65–69 years < 23.3 kg 70–74 years < 21.2 kg 75–79 years < 18.7 kg 80–84 years < 15.7 kg 85 years and older < 13.2 kg	60–64 years < 14.6 kg 65–69 years < 13.6 kg 70–74 years < 12.4 kg 75–79 years < 11.1 kg 80–84 years < 9.6 kg 85 years and older < 8.8 kg

European consensus EWGSOP2 2018: European Working Group on Sarcopenia in Older People update in 2018^5^. Asian consensus 2019^11^. Chileans 2018: Reference values of handgrip dynamometry in older Chileans^13^. Original Colombian cutoffs 2019: reference cutoffs for handgrip strength among older adults^17^. Alternative Colombian borderlines2019 (< p25): values lower than the 25-percentile taken from Ramirez-Velez et al.^17^.

Gait speed was assessed in a five-meter walk on a flat surface. In the first meter, the participants assessed were allowed to reach a regular walking pace, and in the last meter, the participants were allowed to slow down. The walking time from the beginning of the second meter up to the end of the fourth meter was recorded with a digital stopwatch^28,29^. When the speed was below 0.8 m per second, the participant was classified with a slow gait speed^30^.

Face-to-face interviews were used to collect data on sociodemographic variables, health conditions, and physical activity. The Advanced Activities of Daily Living scale by Reuben et al. was used to classify people as active and inactive^31^. The Mini Nutritional Assessment was applied to classify people with malnutrition (< 17 points), at risk of malnutrition (17–23.5 points), or under normal nutritional status (≥ 24 points)^26^. Risk of malnutrition and malnutrition categories were combined due to the low frequency of malnutrition (n = 34). Likewise, BMI was calculated, and the older people were classified using the Pan American Health Organization cutoffs for underweight, normal weight, overweight, and obesity^32^. Information about the participants’ diagnostic diseases was obtained by a physician and registered using the most prevalent list. Suffering two or more medical conditions/diseases was classified as multimorbidity, following the WHO guidelines^33^.

The statistical analysis was done using SPSS software version 25 (Chicago: SPSS Inc.; Ill). The quantitative variables were checked for normal distribution using the Kolmogorov–Smirnov test. These variables are described with median and interquartile range (IQR). According to the data distribution, the Mann–Whitney U or Kruskal–Wallis tests were used for comparing handgrip values with demographic, anthropometric, physical activity, and health status variables. Multiple comparisons among groups were done using Dunn´s post-hoc test. Categorical variables are presented as frequencies and percentages. The maximum likelihood test was used to determine their association with the dynapenia classifications. McNemar’s test was used to compare the results of the dynapenia classifications. Cohen’s Kappa coefficient was used to assess concordance between them. Kappa coefficients were interpreted using Landis and Koch; κ values (a) above 0.80 indicated an almost perfect agreement, (b) from 0.61 up to 0.80 indicated substantial agreement, (c) from 0.41 up to 0.60 indicated moderate agreement, (d) from 0.21 up to 0.40 indicated fair agreement, and (e) between 0.00 and 0.20 indicated a slight agreement^34^. Bangdiwala charts were built to visualize agreement analysis of the dynapenia classifications using a vcd package in R^35^. A perfect agreement is determined when the black squares and the rectangular boxes of the diagonal chart have the same size. The disagreement increases as the black square's size decreases compared to the cells' rectangular area. Bias increases positively or negatively in accordance with the vertex line connecting the rectangles, when pointing up or down the diagonal. A full explanation of Bangdiwala’s agreement chart can be found somewhere else^36^. *P*-values < 0.05 were considered as significant.

## Supplementary Information

Supplementary Information.

## Data Availability

The data that support the findings of this study are available from Gobernación de Antioquia-Colombia (*Gerencia de Seguridad Alimentaria y Nutricional* office), but restrictions apply to the availability of these data, which were used under license for the current study, and so are not publicly available. Data are however available from the authors upon reasonable request and with permission of *Gobernación de Antioquia-Colombia* (*Gerencia de Seguridad Alimentaria y Nutricional* office).
