# Supplementary material for: Handgrip strength in older adults from Antioquia-Colombia and comparison of cutoff points for dynapenia
Source: Sci Rep. 2023 Jan 31;13:1718. doi: 10.1038/s41598-023-28898-1 (PMC9889798; doi:10.1038/s41598-023-28898-1)
Supplement: Supplementary file 1 — Supplementary Information. [file 41598_2023_28898_MOESM1_ESM.docx]

**Supplementary Table S1. Concordance between different cutoff points for dynapenia according to sex.**

|  | | **Men** | | | **Women** | | |
| --- | --- | --- | --- | --- | --- | --- | --- |
|  |  | **Original Colombian cutoffs 2019** | | **Kappa** | **Original Colombian cutoffs 2019** | | **Kappa** |
|  |  | **Normal** | **Dynapenia** |  | **Normal** | **Dynapenia** |  |
| European consensus (EWGSOP) 2018 | Normal | 524 | 0 | K=0,049; p=<0,001 | 749 | 0 | K=0,068; p=<0,001 |
|  | Dinapenia | 150 | 5 |  | 157 | 7 |  |
| Asian Consensus 2019 | Normal | 494 | 0 | K=0,039; p=<0,001 | 657 | 0 | K=0,039; p=<0,001 |
|  | Dinapenia | 180 | 5 |  | 249 | 7 |  |
| Chileans 2018 proposal | Normal | 494 | 0 | K=0,039; p=<0,001 | 749 | 0 | K=0,068; p=<0,001 |
|  | Dinapenia | 180 | 5 |  | 157 | 7 |  |
| K: Cohen’s Kappa Coefficient. McNemar: McNemar test. P: p value. | | | | | | | |

|  | | **Men** | | | **Women** | | |
| --- | --- | --- | --- | --- | --- | --- | --- |
|  |  | **Alternative Colombian borderlines 2019 <p25** | | **Kappa** | **Alternative Colombian borderlines 2019 <p25** | | **Kappa** |
|  |  | **Normal** | **Dynapenia** |  | **Normal** | **Dynapenia** |  |
| European consensus (EWGSOP) 2018 | Normal | 524 | 0 | K=0,349; p=<0,001 | 749 | 0 | K=0,411; p=<0,001 |
|  | Dinapenia | 115 | 40 |  | 115 | 49 |  |
| Asian Consensus 2019 | Normal | 494 | 0 | K=0,286; p=<0,001 | 657 | 0 | K=0,254; p=<0,001 |
|  | Dinapenia | 145 | 40 |  | 207 | 49 |  |
| Chileans 2018 proposal | Normal | 494 | 0 | K=0,286; p=<0,001 | 749 | 0 | K=0,411; p=<0,001 |
|  | Dinapenia | 145 | 40 |  | 115 | 49 |  |
| K: Cohen’s Kappa Coefficient. McNemar: McNemar test. P: p value. | | | | | | | |
|  | | | | | | | |

*European consensus EWGSOP2 2018: European Working Group on Sarcopenia in Older People update in 2018^5^. Asians consensus 2019^11^. Chileans 2018: Reference values of handgrip dynamometry in older Chileans^13^. Original Colo*m*bian cutoffs 2019: reference cutoffs for handgrip strength among older adults^17^. Alternative Colombian borderlines2019 (< p25): values lower than the 25-percentile taken from Ramirez-Velez et al. ^17^.*
